# A progressive sclerotic depigmenting congenital melanocytic nevus in a Chinese patient^؆^

**DOI:** 10.1016/j.abd.2026.501341

**Published:** 2026-04-28

**Authors:** Yufan Chen, Yuqing Wang, Juanmei Cao, Tianqi Zhao, Jing Yang, Changzheng Huang

**Affiliations:** a Department of Dermatology, Union Hospital, Tongji Medical College, Huazhong University of Science and Technology, Wuhan, Hubei, China; b Department of Clinical Nutrition, Union Hospital, Tongji Medical College, Huazhong University of Science and Technology, Wuhan, Hubei, China; c Department of Dermatology, First Affiliated Hospital, Shihezi University, Shihezi, Xinjiang, China

Dear Editor,

Congenital Melanocytic Nevi (CMN) present in approximately 1%‒2% of newborns.^1^ They are usually brown to black, hairy lesions with a consistency similar to that of the surrounding normal skin. CMN is classified according to the diameter of the lesion: <1.5 cm is classified as Small Congenital Melanocytic Nevi (SCMN), 1.5‒20 cm as Medium Congenital Melanocytic Nevi (MCMN), 20‒40 cm as Large Congenital Melanocytic Nevi (LCMN), and >40 cm as Giant Congenital Melanocytic Nevi (GCMN).

Spontaneous regression of medium-to-large congenital melanocytic nevi is rare, but regression presenting as depigmentation with sclerosis, alopecia, or pruritus is exceedingly so.

Herein, we present a 15-month-old Chinese child with progressive regression of pigmentation and hardness of a hairless pruritic medium-sized CMN. This male infant represents the first reported case in China of progressive sclerosing hypopigmented Congenital Melanocytic Nevus (CMN). The lesion has been present on his occipital scalp since birth. At 1-month of age, the lesion presented as a hard, slightly raised, black plaque measuring 10 × 8 cm, with a hypopigmented area in the upper left quadrant (Fig. 1A).

**Fig. 1:**
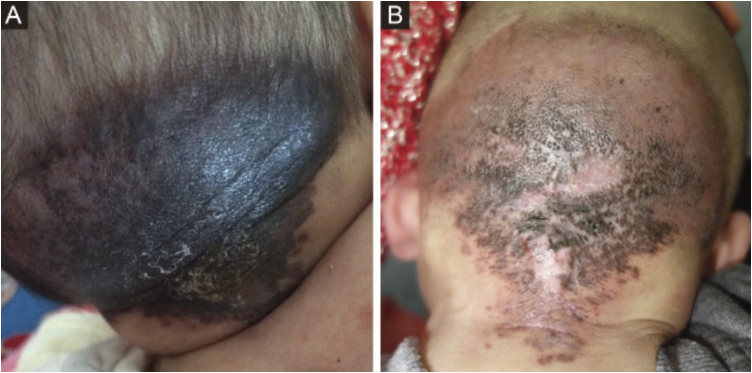
(A) Lesion at 1-month of age: A 10 × 8 cm, hard, slightly raised black plaque with a hypopigmented area in the upper left quadrant. (B) Lesion at 15-months of age: A 14 × 11 cm, hardened, hairless surface with irregular dark-brown pigmentation and a bordered area of mild erythema.

As he grew older, the lesion exhibited progressive changes: hypopigmentation, sclerosis, hair loss, and pruritus. By 15-months, it had expanded to 14 × 11 cm, featuring a hardened, hairless surface with irregular dark-brown pigmentation and a bordered area of mild erythema. The periphery was accompanied by small-sized, scattered lesions and areas of hypopigmentation (Fig. 1B).

Histopathology demonstrated nests of melanocytes in the dermoepidermal junction and superficial dermis with markedly reduced skin appendages (Fig. 2A); the reticular dermis exhibited abundant collagen bundles and fibroblasts surrounding the nevus cell nests (Fig. 2B).

**Fig. 2:**
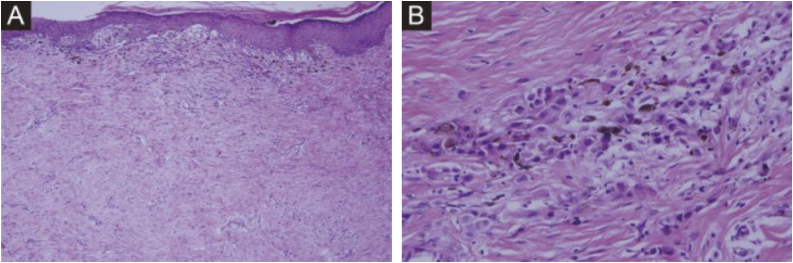
(A) Nevus cell nests in the superficial dermis (Hematoxylin & eosin, ×100). (B) Collagen bundles and fibroblasts surrounding nevus cell nests (Hematoxylin & eosin, ×400).

Immunohistochemistry (Fig. 3A‒D) confirmed nevus cell positivity for S100, Melan-A, and HMB45, negativity for p53, and a Ki-67 proliferation index <10%. Both biopsied lymph nodes showed reactive hyperplasia.

**Fig. 3:**
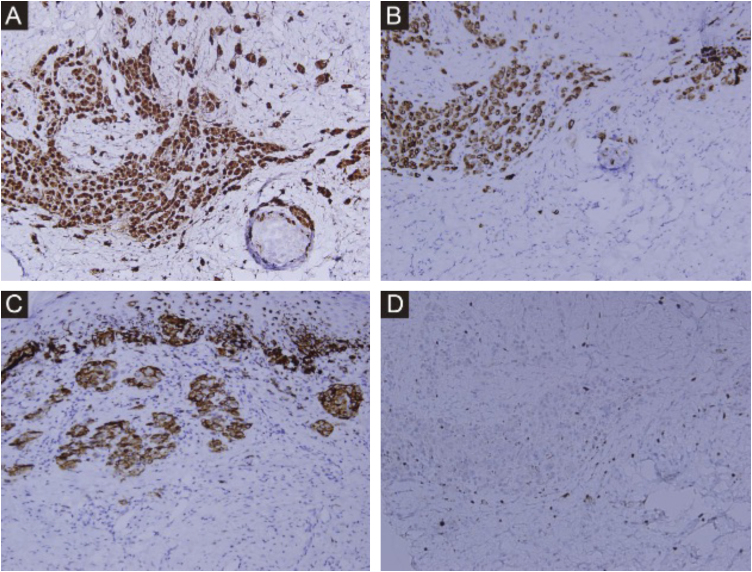
Immunohistochemical staining (A) S100 Staining, ×200. (B) Melan-A Staining, ×200. (C) HMB45 Staining, ×200. (D) Ki-67 Staining, ×200.

PubMed was searched, and 13 cases of spontaneous regression of CMN with sclerosis. This cohort comprised eight females and five males. The onset age ranged from birth to three years, and the depigmentation process lasted less than eight years. The lesions were most commonly present on the trunk (10/13), then were found on the scalp (2/13), feet, and ankles (1/13). All the lesions were >1.5 cm in diameter. Sclerosis and pigmentation regression occurred in all 13 cases. Hair loss (9/13), pruritus (6/13), and ulcer (4/13) were also observed. The mechanism for these has been proposed as an autoimmune reaction. The autoimmune response targeting melanocytes may be responsible for the regression of pigmentation. Meanwhile, this autoimmune response would produce large amounts of inflammatory factors that induce collagen proliferation, resulting in sclerosis.^2^ There was induration in a few cases,^3^, ^4^ but no nodular lesions. Usually, induration denotes local hardening, whereas sclerosis means diffuse hardening. Localized induration on a CMN raises the possibility of malignant transformation, in contrast to the diffuse hardening of sclerotic CMNs, which is considered benign.^4^, ^5^ In our case, we performed immunohistochemistry staining and observed a low proliferation index with Ki-67. In addition, none of the CMNs described so far have progressed to melanoma; the longest recorded follow-up is 17-years.^5^

In conclusion, we reported the first case of progressive sclerotic hypopigmented CMN in a Chinese patient. Continuous monitoring is required for this rare disease, but there is no need for excessive panic and extensive excision due to the low possibility of malignant transformation.

## ORCID ID

Yufan Chen: 0009-0009-6002-0699

Yuqing Wang: 0000-0001-9320-3517

Juanmei Cao: 0000-0002-6077-2585

Tianqi Zhao: 0000-0002-9771-9753

Jing Yang: 0000-0001-9882-4697

Changzheng Huang: 0000-0003-1531-1269

## Financial support

This work was supported by Science and Technology Program of XPCC (2024ZD042, 2023ZD023).

## Authors' contributions

Yufan Chen: Preparation and writing of the manuscript; critical literature review; data collection, analysis and interpretation.

Yuqing Wang: Preparation and writing of the manuscript; data collection, analysis and interpretation.

Juanmei Cao: Data collection, analysis and interpretation; final approval of the final version of the manuscript.

Tianqi Zhao: Data collection, analysis and interpretation; final approval of the final version of the manuscript.

Jing Yang: Approval of the final version of the manuscript; effective participation in research orientation; final approval of the final version of the manuscript.

Changzheng Huang: Approval of the final version of the manuscript; effective participation in research orientation; final approval of the final version of the manuscript.

## Research data availability

All data supporting the findings are included within the article.

## Conflicts of interest

None declared.
